# The Present State of the Use of Waste Wood Ash as an Eco-Efficient Construction Material: A Review

**DOI:** 10.3390/ma15155349

**Published:** 2022-08-03

**Authors:** Rebeca Martínez-García, P. Jagadesh, Osama Zaid, Adrian A. Șerbănoiu, Fernando J. Fraile-Fernández, Jesús de Prado-Gil, Shaker M. A. Qaidi, Cătălina M. Grădinaru

**Affiliations:** 1 Department of Mining Technology, Topography and Structures, Campus of Vegazana s/n, University of León, 24071 León, Spain; rmartg@unileon.es (R.M.-G.); fjfraf@unileon.es (F.J.F.-F.); jesusdepradogil@gmail.com (J.d.P.-G.); 2 Department of Civil Engineering, Coimbatore Institute of Technology, Coimbatore 641014, Tamil Nadu, India; jaga.86@gmail.com; 3 Department of Civil Engineering, Swedish College of Engineering and Technology, Wah Cantt 47080, Pakistan; 4 Faculty of Civil Engineering and Building Services, Gheorghe Asachi Technical University of Iași, 700050 Iași, Romania; serbanoiu.adrian@tuiasi.ro (A.A.Ș.); catalina.gradinaru@tuiasi.ro (C.M.G.); 5 Department of Civil Engineering, College of Engineering, University of Duhok, Duhok 42001, Iraq; shaker.abdal@uod.ac

**Keywords:** geopolymer concrete, waste wood ash, environmental impact mechanical properties, durability

## Abstract

A main global challenge is finding an alternative material for cement, which is a major source of pollution to the environment because it emits greenhouse gases. Investigators play a significant role in global waste disposal by developing appropriate methods for its effective utilization. Geopolymers are one of the best options for reusing all industrial wastes containing aluminosilicate and the best alternative materials for concrete applications. Waste wood ash (WWA) is used with other waste materials in geopolymer production and is found in pulp and paper, wood-burning industrial facilities, and wood-fired plants. On the other hand, the WWA manufacturing industry necessitates the acquisition of large tracts of land in rural areas, while some industries use incinerators to burn wood waste, which contributes to air pollution, a significant environmental problem. This review paper offers a comprehensive review of the current utilization of WWA with the partial replacement with other mineral materials, such as fly ash, as a base for geopolymer concrete and mortar production. A review of the usage of waste wood ash in the construction sector is offered, and development tendencies are assessed about mechanical, durability, and microstructural characteristics. The impacts of waste wood ash as a pozzolanic base for eco-concreting usages are summarized. According to the findings, incorporating WWA into concrete is useful to sustainable progress and waste reduction as the WWA mostly behaves as a filler in filling action and moderate amounts of WWA offer a fairly higher compressive strength to concrete. A detail study on the source of WWA on concrete mineralogy and properties must be performed to fill the potential research gap.

## 1. Introduction

Globally, 0.74 kg of solid waste is generated per capita per day, with national rates varying between 0.11 and 4.54 kg per capita per day depending on urbanization rates and income levels [1,2,3]. The Europe and Central Asia regions, with 20% (392 million tons per year), rank second in solid waste generation [4,5,6]. The overall composition of waste mainly corresponds to organic and green waste (44%); paper and cardboard (19%); other materials (14%); plastics (12%); glass (5%), metal (4%); wood (2%); and rubber and leather (2%). As for waste treatment, it mainly focuses on recycling (20%) and incineration (17.8%), providing the possibility of giving a new useful life to the materials after their use and ensuring adequate final disposal [7]. This is in line with the adaptation of a circular economy as a novelty and eco-friendly production model. In the specific case of the construction industry, part of the environmental impact is due to the demolition of structures, which generates different types of solid waste. On the other hand, the use of cement in the production of bricks/block and concrete, which is used in the latter to make it more resistant [8], implies a significant anthropic emission of carbon dioxide (CO_2_) of 5–8% worldwide, which could increase, according to projections, to 27% by the year 2050, especially taking into account that one cubic meter of concrete is produced annually per person [8,9,10,11]. Based on this reality and the projected scenario, the cement and concrete industry has been developing a series of strategies and innovations to reduce CO_2_ emissions. One of these innovations is the production of geopolymers to be used as alternative materials to replace all or part of the ordinary Portland cement used in construction, which is obtained either from metakaolin or from industrial, forestry, and agricultural waste with a high aluminosilicates content [12,13,14,15]. A geopolymer is a binder of mineral origin (inorganic) obtained from the dissolution [16,17,18,19] and subsequent polycondensation of ashes rich in aluminosilicates in the presence of an alkaline solution (hydroxides and silicates of alkali metals, Na and K) [13,20,21]. Additionally, the use of mixed geopolymers, which are generated by the combination of two or more types of chemically stabilized industrial wastes or ashes, has been considered [22]. The use of this type of materials can reduce CO_2_ production by up to 90%, while preserving or even improving their mechanical properties (e.g., porosity, structure, compressive strength, water absorption, and durability) [12,23].

Several researchers have devoted themselves to using different raw materials for the production of concrete, for example, agricultural residues such as rice husk ash and palm oil ash [24], sugar cane bagasse [25,26], and corn cob ash [27], finding good results in the properties of concrete [28]. On the other hand, wood waste ashes [29,30] have emerged as a good option for the fractional replacement of binder and kaolin used in the formation of geopolymers, since in addition to increasing workability, porosity, and drying shrinkage, these wastes are given an alternative use, and potential environmental pollution [29,30,31,32,33] is reduced by their entry into the environment, contributing directly to sustainable development [34,35]. Ekaputri reported [36] obtaining a concrete (geopolymer) with high compressive strength (48.5 MPa to 48.5 MPa) from class F ash with 10 mol/L NaOH due to the generation of hydroxide ions that significantly influence the dissolution of the Si and Al atoms of the source material. Despite the advantages of using high concentrations of alkali (NaOH, between 8–10 M) to obtain a high compression strength product of 104.5 MPa and 71 MPa for the paste and mortar, as well as a lower change in length due to temperature and water evaporation that have the lowest shrinkage percentage [37], it has been proven that the use of ashes from forest biomass (wood) can decrease the requirements of alkaline activators by up to 20% without the loss of properties [38,39,40,41]. However, when the substitution level of these ashes is higher than 10% by mass, the mechanical properties of the geopolymer are affected [42,43,44,45], proportionally reducing the compressive and flexural strength of the mortars, for all curing times [35]. Likewise, it is highlighted that different conditions can be used during the process of obtaining geopolymers, such as the type of curing, humidity control, temperature, concentration and proportions of alkaline activators, type and quantity of raw material or proportions of starting materials (in case of mixtures), which will influence the properties of the final product. Among the findings, it can be mentioned that the increase in SiO_2_/Al_2_O_3_ ratios positively influences the mechanical compressive strength of geopolymers [15], and it was found that the inclusion of 5–15% wood ash in the process can generate greater strength and durability depending on the age (aging time) of 3–7 days as a consequence of the formation of gels and minerals that increase alkalinity [12]. Research has also been conducted on the effects of the solid–liquid ratio and the alkaline activator in the synthesis of pure geopolymers. Alves et al. [46] used as precursor material ground blast furnace slag with a solid–liquid ratio between 1.5 and 2.2, and as activator solutions (a) a sodium hydroxide/sodium silicate/water mixture and (b) a potassium hydroxide/potassium silicate/water mixture, finding that the resulting geopolymer possessed high compressive strength depending on the solid–liquid ratio and the percentage of water added to the mixture, which is further impacted by the composition of the activating solution. They also noticed that the strength increases with aging [46]. Currently, the addition of plastics to the optimized wood ash-based geopolymer is being tested; for example, in the case of polypropylene (PP), it has been reported that the addition of 1% PP fiber generates an increase in compressive, tensile, and flexural strength by 3.7%, 15.6%, and 10%, respectively [47]. Other types of materials are also being developed. Kristály et al. [48] produced a composite of geopolymer foam and glass to obtain a lightweight and environmentally friendly concrete from waste materials (secondary raw material), which is a valuable building material useful for thermal and acoustic insulation of walls that is also heat-, fire-, and acid-resistant [48].

Cement consumption in the world currently amounts to approximately 3 billion tons, which translates into 1.5 billion tons of carbon dioxide emitted into the environment [34]. According to the United Nations, the world population has increased in recent years, from 5300 million inhabitants in 1990 to 7300 million inhabitants in 2015; with a projected increase by the year 2050 of 24.74%, the requirements for cement, concrete, and other types of construction materials will increase significantly [49]. In this sense, the development of new and better alternative materials for the efficient substitution of cement for other materials at a global level will reduce production costs while reducing emissions, contributing to goals 11 and 13 of the 2030 Agenda for Sustainable Development, “Make cities and human settlements inclusive, safe, resilient and sustainable” and “Climate action”, respectively. This literature review focuses on the approach to the processes for obtaining geopolymers from the use of wood ash, as well as the physical and chemical effects that take place under different production conditions. As per the authors’ best knowledge, no significant review study exists on the physical, chemical, strength, durability, and microstructural analysis of concrete, which points to the originality of present work.

## 2. Environmental Impact of WWA

### 2.1. Air Pollution

Energy extracted from burning the wood results in the formation of WWA. WWA is very fine, which results in the ease of pollution causing respiratory problems for human beings and animals around the site of WWA production [50]. Loose ash has a high possibility for harmful influence on ground vegetation [51], predominantly to the cover and certain kinds of moss groups [52].

### 2.2. Land Pollution

WWA is problematic if spread regularly and requires slow delivery rates from spreaders [51]. Because of the huge variety of WWA quality, reliance on the sort of chemical structure of WWA is needed before demonstrating the direction of management as agricultural or forest-related systems [53]. WWA recycling to agricultural or forests appears a decent environmental solution, but there are a lot of possible difficulties related to its use in systems, which are more multifaceted [51].

### 2.3. pH Increase

The topsoil of the system is affected by pH differences and its blocks the crop or tree to obtain enough amount of nutrition from the soil. The delivery rate of calcium to soils is reliant on the primary shape of the ash, with loose ash such as WWA possibly instigating a temporary quick increase in pH in the soil [54]. For the first 7 years, the soil under 100 mm depth had a very minor change in pH value after WWA application, but after 16 years, an increase in pH value was observed [51]. The land dumping of WWA results in the slow transfer of pH from the topsoil to bottom soil, which can be observed over time. There is an increase in the pH of runoff water over the same period where WWA is applied, as observed by Fransman et al. [55].

### 2.4. Higher Production Rate

Approximately 2.5 kilo-tons of WWA are annually discarded in lands, as of 2006 [51], but it may increase at a high rate and a decrease in forest land has been observed. In several countries across the globe, 90% of WWA is sent to landfill and the balance part goes as land applied purpose, co-composed with sewage sludge [51]. Apart from the several environmental effects discussed above, Pitman et al. (2006) [51] studied specifically soil properties and soil vegetation.

### 2.5. PH Affects the Nutrition (Phosphorus, Nitrogen, and Potassium) Addition of Soil

When WWA is in contact with water, the pH solution becomes higher as the hydroxides and oxides in the WWA are dissolved and hydroxide ions are developed. WWA has a liming impact when introduced into soils and could be utilized to neutralize acidity. Three tons of WWA have a liming effect equal to one ton of quicklime. The solubility of different nutrition elements in the WWA varies considerably. Generally, the solubility of the nutrients elements are in the order of potassium > magnesium > calcium oxide > phosphorus [56].

### 2.6. Heavy Metal Contamination of Soil

pH, organic material content, and hydrous oxide play the main roles in the adsorption of heavy metals from the soil [57]. When WWA dissolves in an acid environment such as soils in forests, the alkalinity of WWA is consumed and the metals are exposed to a pH far lower than that of the ash, causing higher solubility [56]. WWA could also have high concentrations of heavy metals due to the fuel, which is contaminated. Wood from and wood preservers and demolition in waste wood generally comprise higher proportions of heavy metals. As a result of the relatively low volatilization temperatures for many of the heavy metals, they become enhanced by WWA. In the combustion of untreated wood [58], the concentrations of lead and antimony are one order of magnitude higher, while the concentrations of arsenic, cadmium, chromium, copper, nickel, and zinc are approximately twice as high.

### 2.7. Soil Water Leachate

Williams et al. (1996) [59] observed that there are amplified concentrations of both calcium and potassium in groundwaters and soils, with some movement of aluminum and magnesium in it. In a long-term experiment, soil leachate at a 20 cm depth taken from mineral soils displayed elevated levels of calcium, magnesium, and potassium, but no significant impact on nitrate concentration, pH or Cd, Cu, Cr, and Pb levels [51]. The storage of moistened WWA in the air led to an adverse effect: it increased potassium leaching. The leaching of phosphorous, magnesium, and metal species from the ash matrix is generally low with a high pH prevailing in the water phase during short-term leaching [60].

## 3. Source and Production of WWA

As per Grau et al. [61], the WWA obtained from the total amount of available quantity is from 0.4 to 2.1%.

## 4. Physical and Chemical Compositions

### 4.1. Physical Properties

As per the report from Etiegni and Campbell et al. [62], 80% of WWA consists of particles size less than 1.0 mm and the balance is unburned wood particles with different sizes. Specifically, WWA consists of 25.4% of fine particles, which are less than 75 µm. Wood ash shows a relatively high specific surface area that provides good absorption. The specific gravity of WWA is 2.41, pH 12.57, average particle diameter d_50_ (mm) is 0.223, bulk density (kg/m^3^) is 663–997, and specific surface area (m^2^/kg) is 4200–100,600 (Grau et al., 2015) [61]. An increase in the particle size increases the concentrations of aluminum, arsenic, barium, and copper, and decreases the concentrations of boron, cadmium, manganese, lead, zinc, potassium, magnesium, and calcium [63].

### 4.2. Chemical Properties

Wood combustion produces highly alkaline ash (pH varies from 9 to 12) [62]. Ash yield is decreased approximately to 45% with an increase in burning temperature from 538 °C to 1093 °C. The metal contents in ash increase with the increase in the burning temperature. With the increase in the burning temperature, elements such as calcium, iron, magnesium, manganese, and phosphorus increase, and elements such as zinc, potassium, and sodium decrease [62].

The alkalinity of WWA depends on the carbonate, bicarbonate, and hydroxide content in it. WWA composition also varies during storage and under different environmental conditions as carbon dioxide and moisture react with WWA to form carbonates, bicarbonates, and hydroxides [62]. An increase in potassium, sodium, and manganese concentration increases linearly with ash concentration. The leaching of these elements increased to result in a decrease in pH. Table 1 shows the chemical elements of different types of ashes.

Zajac et al. [53] divided ash components into three categories based on their concentration: 1. macro-elements: phosphorus, potassium, calcium, and sulfur; 2. micro-elements: manganese, iron, copper, and zinc; 3. toxic elements: chromium, nickel, arsenic, and lead. The quantity and quality of WWA content depend upon the organic, inorganic, and impurity elements present in it. The chemical and physical characteristics of WWA depend upon on the sampling point, the sort of biomass, plant kind, growth process, growth circumstances, plant age, fertilization, the applied dosage of plant protection products, harvesting conditions, and process of burning (preparation of fuel, burning method used, and circumstances) [51,53].

Szakova et al. [64] determined the chemical composition of WWA (wood chips and wood waste) using the XRF technique. Different elements analyzed (in ppm) were P: 5300–10,800; S: 1200–11,100; K: 38,000–58,000; Ca: 78,000–159,000; Cr: 118; Mn: 6200–10,700; Fe: 29,300–34,800; N: 28.9; Cu: 153; Zn: 300–1100; As: 9.8; and Pb: 313. Tarun et al. (2003) revealed the subsequent elements in wood ash: C (5% to 30%), Ca (5% to 30%), carbon (7% to 33%), K (3% to 4%), Mg (1% to 2%), P (0.3% to 1.4%), and Na (0.2% to 0.5%). Elemental arrangement varies for WWA because ashes derived from branches and roots are rich in many elements than those derived from stem wood [51].

The following compound composition limits were also reported: titanium dioxide (0% to 1.5%), sulfur trioxide (0.1% to 15%), silica (4% to 60%), aluminum oxide (5% to 20%), ferric oxide (10% to 90%), magnesium oxide (0.7% to 5%), potassium oxide (0.4% to 14%), calcium oxide (2% to 37%), loss of ignition (0.1% to 33%), moisture content (0.1% to 22%), and available alkalis (0.4% to 20%). Table 2 shows the chemical compounds in waste wood ash that were obtained in past research.

WWA is usually very low in nitrogen because it evaporates during incineration. Trace elements such as boron (B), molybdenum (Mo), copper (Cu), and zinc (Zn) have been observed in WWA, which are called micronutrients [65].

**Table 2:** Chemical compounds of WWA from past studies.

SiO_2_	Al_2_O_3_	Fe_2_O_3_	CaO	MgO	K_2_O	NaO	L.O. I	Ref.
31.8	28	2.34	10.53	9.32	10.38	6.5	1.13	[66]
32.8	27.0	2.2	11.7	9.1	10.5	6.7	0.7	[61]
30.4	26.5	1.9	12.8	9.4	11.4	5.9	1.7	[67]
32.4	27.4	2.1	10.3	9.4	10.85	6.4	1.15	[68]
31.8	28.2	2.4	10.6	9.2	10.80	6.0	1.00	[69]
33.6	27.8	2.6	11.2	8.3	10.2	5.7	0.60	[70]
32.7	26.4	2.2	10.25	9.45	10.45	7.21	1.34	[71]
31.5	26.3	2.6	10.4	9.3	10.72	8.2	0.98	[72]

## 5. Influence of Waste Wood Ash on Hardened Concrete

### 5.1. Strength Properties

Waste wood ash is an easily available agricultural discarded material that enhances the workability, quality of microstructure, and improves the strength characteristics of concrete samples. The key characteristics of concrete can be enhanced, and most importantly, the time for hydration is reduced due to the pozzolanic effect [70]. The distinct proportioning of the mix can be achieved by substituting cement with waste wood ash and slag and other supplementary cementitious materials (SCMs). The different characteristics of concrete from the previous literature that were assessed through different forms of strength and durability testing are presented in Table 3.

The properties of various mixes have been studied with different water to binder ratios. The optimal dose was 20% WWA with rice husk ash, which improved the compression strength significantly and also showed enhanced durability [73]. Because of the low amount of silica in waste wood ash, a low water to cement ratio of 0.4 was selected. Ramos et al. noted that waste wood ash seemed a potential fractional substitute pozzolanic material for cement because it improves strength and durability characteristics and also assists in making concrete sustainable [68].

**Table 3:** Influence of waste wood ash on the characteristics of concrete.

Level of Substitution	Observed Properties	Results	Discussion	Ref.
10–35% (25% optimum dosage)	Specific gravity Bulk density Initial setting time Final setting time Compression strength Slump value Water demand	2.21 755 kg/m^3^ 221 min 547 min 7.5–23.2 MPa at 56 days 40–55 mm 134–140 mL	When the water to cement ratio was kept at 0.55, the maximum slump was 55 mm for 25% and 30% WWA, and maximum strength was 23.2 MPa for 25% WWA at 56 days and then reduced	[66]
0–25% (20% optimum dosage)	Compression strength Water absorption Weight loss	40–48 MPa at 90 days (before acid test), 29–41 MPa (after acid test) 2.2%–2.64% 6–10.5%	When the water to cement ratio was kept at 0.45 and utilizing 10% sulfuric acid, the highest loss in strength was 29 MPa 90 days for; 20% WWA loss in weight was minimum with only 6%	[74]
5–25% (15–20% optimum dosage)	Compression strength Flexural strength Slump value Water absorption	17 MPa to 29 MPa at 28 days 4 MPa to 6.25 MPa at 7 and 28 days 0–15 mm 0.20% to 1.70%	At a water to binder ratio of 0.50, samples with 15 and 20% WWA had maximum compressive strength with 17 and 29 MPa at 28 days, and then strength began to decrease	[67]
10–30% (20% optimum dosage)	Split tensile strength Compression strength	4.25 MPa to 6.70 MPa at 28 and 90 days 42 to 49 at 28 days and 47 to 55 MPa at 90 days	At a water to binder ratio of 0.48, at 20% WWA, the strength was slightly less than the reference sample due to WWA acting as a filler, not a binder, but microstructure was enhanced	[72]
0–20% (20% optimum dosage)	Compression strength Flexural strength Alkali silica reaction Carbonation	39 to 54 MPa at 28 to 90 days 7 to 9 MPa at 56 days Expansion of ASR at 28 days was 0.17% at 20% WWA The average depth was 3.75 mm at 20% WWA	The highest compression strength was obtained at 54 MPa at 90 days with water to cement ratio of 0.52 with 20% WWA; the same w/c led to a sample with low ASR levels and carbonation depth	[68]
0–40% (25% optimum dosage)	Compression strength Slump value	12–15 MPa at 21 days without admixture, with admixture the strength 28 MPa, With w/c 0.55, the slump was 40 mm	Sample with w/c of 0.55 had the highest slump value 40 mm at 15% WWA, utilizing admixture enhanced compression strength significantly with 45% more strength at 25% WWA	[75]
10–25% with 5% silica fume (20% + 5% SF optimum dosage)	SEM analysis Compression strength	The creation of pores in mortars was considerably impacted because of the substitution of the binder with WWA and SF 20–42 MPa at 28 days	With constant w/c of 0.44, mix with 20% WWA and 5% SF had the highest mechanical strength, and further adding of WWA led to the development of pores in the matrix	[76]
10–35% (20% optimum dosage)	Compression strength Compaction factor	29.5–54 MPa at 90 days 0.741	At later ages, the concrete strength improved considerably, because the water absorption from the blend by WWA reduced the workability slowly	[77]
0–30% (25% optimum dosage)	Pressure resistance Water absorption	2.9–3.8 9–11.5	With a 25% dose of WWA, the samples had the least absorption and maximum pressure resistance in comparison to the reference sample.	[78]
0–25% (20% optimum dosage)	Slump value Compression strength Sieve analysis	45 mm with 20% WWA 10.57 to 35.47 MPa at 20% WWA for 28 days Size ranged from 0.059 to 32.5 mm	With a w/c of 0.55, the optimal mechanical strength was 35.4 MPa at 90 days with 20% WWA as a partial substitute for cement, and workability was in an acceptable range	[79]
5–20% (15% optimum dosage)	Chemical and physical analysis Compression strength Flexural strength Split tensile strength X-ray diffraction spectra	Comprised 70.5% silica, alumina, and ferric that was similar to class F type pozzolanic material and mean size, bulk density, and specific gravity of WWA were 170 microns, 720 kg/m^3^, and 2.21, respectively For w/b of 0.40, the strength was 36.3 MPa at 28 days For w/b of 0.40, the strength was 6.52 MPa at 28 days For w/b of 0.40, the strength was 2.37 MPa at 28 days WWA comprised silica both in crystal and formless shapes with the highest peak at 29 degrees against 2-theta	At a w/b ratio of 0.40, with 15% of waste wood ash as a partial substitute of cement, the highest compression at 28 days was 36.3 MPa, which was more than the control sample	[80]

### 5.2. Effect of Waste Wood Ash (WWA) on the Durability Characteristics of Concrete

#### 5.2.1. Acid Resistance Test

Dashibil and Udoeyo (2002) [81] examined the capability of waste wood ash concrete to withstand acid tests. Two groups of samples had a similar content of aggregates, water, and binder. The only difference was that the first group had only cement as the primary binder and the other group had 15% waste wood ash as a fractional binder substitute. The concrete samples were dipped in strong acid (sulfuric acid) for 54 days. It was revealed that concrete samples that had waste wood ash had a less decrease in their mass loss in comparison to concrete with no waste wood ash at all.

**Figure 1:**
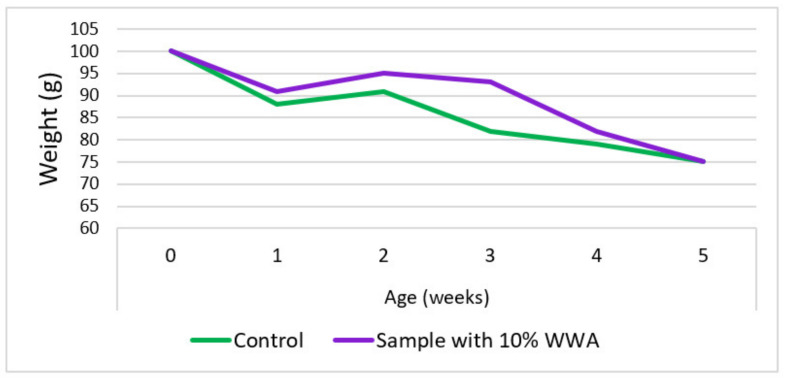
Change in concrete mass with a period of dipping samples in nitric acid (data from reference [82]).

**Figure 2:**
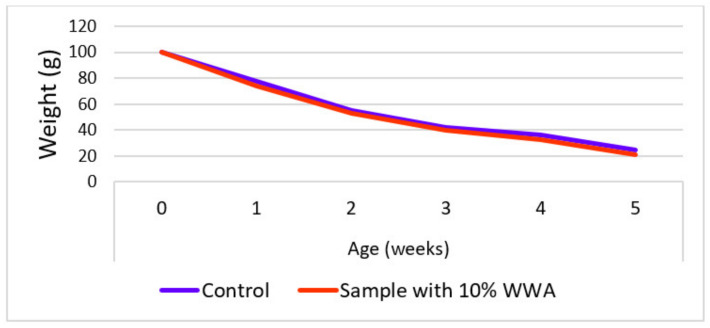
Change in concrete mass with a period of dipping samples in sulfuric acid (data from reference [82]).

Ejeh and Elinwa (2004) [82] investigated the impact of adding WWA in samples for acid tests against the possibility of corrosion. Two sorts of acids were tried; one was sulfuric acid and the other was nitric acid at 20% concentration. One group of samples had 10% WWA utilized as a fractional binder substitute and the other group of samples was the same as the previous mixes but without WWA. Both groups of samples were dipped in both sorts of acids for 35 days. It was noticed that, in samples with 10% WWA, their resistance to nitric acid was much more enhanced because the loss in mass was lower in comparison to the samples with no WWA, as shown in Figure 1. However, samples with 10% WWA had less resistance to sulfuric acid in comparison to samples with no WWA. This is because of a higher loss in the weight of 10% WWA concrete in comparison to the control sample when dipped in 20% H_2_SO_4_, as shown in Figure 2.

#### 5.2.2. Water Absorption

Ejeh and Elinwa (2004) [82] inspected the influence of the inclusion of WWA as a fractional binder substitute in mortar blends on the properties of water absorption. Two groups of mortars were developed with similar mixing content except for cement; one blend had only cement as a binder and the other one had 15% WWA as a fractional substitute of cement in the blend. It was revealed that the addition of WWA as a binder substitute at 15% of cement weight assisted in decreasing the water absorption of the developed blends. The mean water absorption of the blends with 15% WWA and no WWA proportions were noted to be 0.75% and 1.30%, correspondingly, but both of the blends were still lower than 10% of the highest water absorption criteria.

**Figure 3:**
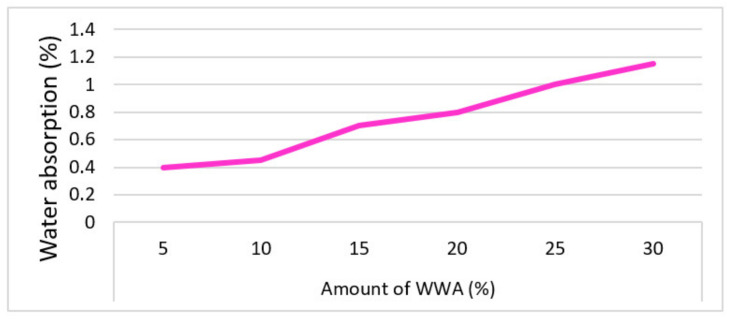
Co-relation of water with WWA in concrete (data from reference [82]).

Udoeyo et al. (2006) [67] studied the characteristics of water absorption with WWA as a fractional binder substitute material. Sample blends with proportions of WWA ranging from 5 to 30% at an interval of 5% were developed to evaluate the water absorption properties. The water absorption of the sample with WWA as a fractional binder substitute was noted to rise steadily from 0.15 to 1.10% with a rise in the proportion of binder substitution from 5% to 30%, as displayed in Figure 3. At proportions of binder substitution by WWA up to 30%, the developed sample had still reasonable values of water absorption under 10%, which is a tolerable criterion for all of the materials that are used for construction.

#### 5.2.3. Permeability of Chloride Test

Wang et al. (2008) [83] examined the resistance against the permeability of chloride of air entrained in the sample with a fractional substitution of the binder with wood/coal fly ash (WCFA) and wood fly ash (WFA). Proportions of binder substitution by different sorts of FA were utilized as a fractional substitution of binder, such as Class F fly ash, class C fly as, ash from the combusted wood, and coal fly ash. All the concrete specimens were placed in water for 56 days before placing in the chloride permeability test, and the chloride permeability test was conducted per ASTM C 1202 [84]. From the test outcome, it was revealed that the inclusion of WWA at a 25% substitution of cement in the sample had no adverse effect from the chloride on the concrete. The usage of class F/coal mixed and wood ash in fractional replacement of cement had considerable help in dropping the permeability of chloride property of the sample. A minor rise in the permeability of chloride in the sample mix with 25% WWA as cement substitute was noted in comparison to the control sample, perhaps ascribed to the coarse size of WWA particles (30 to 130 microns).

**Figure 4:**
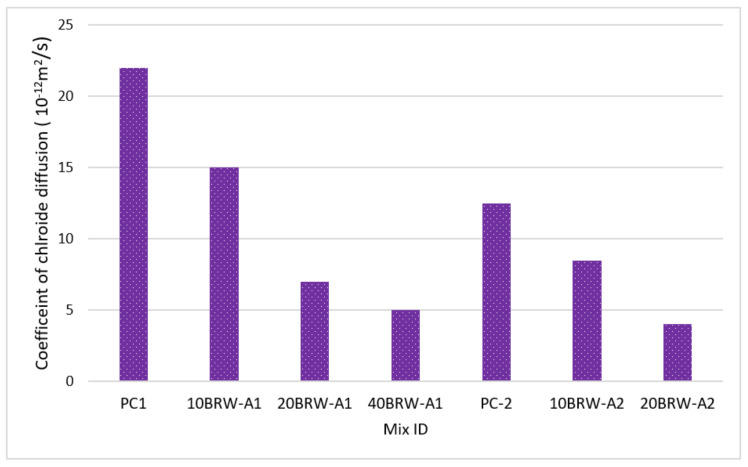
Coefficient of the chloride diffusion of samples at 28 days (data from reference [82]).

Horsakulthai et al. (2010) [73] investigated the impact of adding very fine ash from the combustion of rice husk, wood, and sugarcane waste from bagasse as a fractional binder substitute on the permeability of chloride of a developed blend of concrete. To assess the concrete permeability of chloride, an accelerated salt ponding technique was utilized for two distinct grades of concrete (grades 20 and 35) developed by the inclusion of ash from rice husk, wood, and bagasse at cement substitution proportions of 0, 10, 20, and 40% of cement weight. The test outcome revealed that the inclusion of fine size ash from rice husk, wood, and bagasse as a fractional replacement of binder in the sample led to the improvement in permeability against chloride and also reduced the coefficient of chloride diffusion. The existence of fine size ash from rice husk, wood, and bagasse in a blend at binder substitutions of 10, 20, and 40% caused a decrease in the coefficient of chloride diffusion by 35–45%, 65–75%, and 80% correspondingly, as compared to the reference mix with only Portland cement as a binder. The inclination of a steady decrease in the coefficient of chloride diffusion for the two distinct grades of concrete was evaluated. The raising dose of binder substitution by ash from rice husk, wood, and bagasse is displayed in Figure 4. The term “PC” in Figure 4 denotes plain concrete; BRWA denotes co-combination of bagasse, rice and waste wood ash; and the numbers after them denotes their percentage added in the mix.

#### 5.2.4. Alkali Silica Reaction (ASR)

Baxter and Wang (2007) [85] studied the conduct of expansion in mortar blends due to ASR comprising an opal aggregate, which is a very reactive, highly alkaline cement, and three distinct sorts of fly ash (FA). The different sorts of FA were acquired from heating the class C coal. Four groups of mortar blends with the same proportions of ingredients were arranged. The first group of mortar had only Portland cement as a binder and the remaining three groups of mortar blend had three distinct sorts of FA utilized at a uniform dose of binder substitution with 35% of cement by weight. The test outcome revealed that coal fly ash had a higher quantity of alkaline matter as compared to class C FA. The utilization of coal FA in the blend of mortar was revealed to be capable of decreasing the expansion of the alkali-silica reaction at 180 days under 0.1% (highest expansion stated by ASTM C33) from 0.27%. This happened with the reference blend of mortar having Portland cement as the only binder. Between the different sorts of fly ash that were tested, coal fly ash was revealed to have optimal behavior in the mitigating expansion of the alkali-silica reaction.

#### 5.2.5. Shrinkage Test of WWA Concrete

Naik et al. (2003) [86] examined the dry shrinkage characteristics of sample mixes developed by the inclusion of WWA as a fractional binder substitution material. For blends developed in the research, WWA was utilized at cement replacement levels of 0, 5, 8, and 12%. A variation in length in the formed concrete samples was observed up to 240 days. It was revealed that the value of shrinkage for the reference concrete samples was 0.009% for 7 days and 0.05% for 240 days. The values of shrinkage for sample mixes with 5, 8, 12% were observed to be 0.01–0.02%, 0.0139–0.014%, and 0.049–0.044%. From the test outcomes of dry shrinkage, it was noted that the incorporation of WWA considerably helped in the decrease in the extent of concrete upon drying. This is an essential property that could reduce the development of micro-cracks within the sample upon drying.

## 6. Microstructural Study

**Figure 5:**
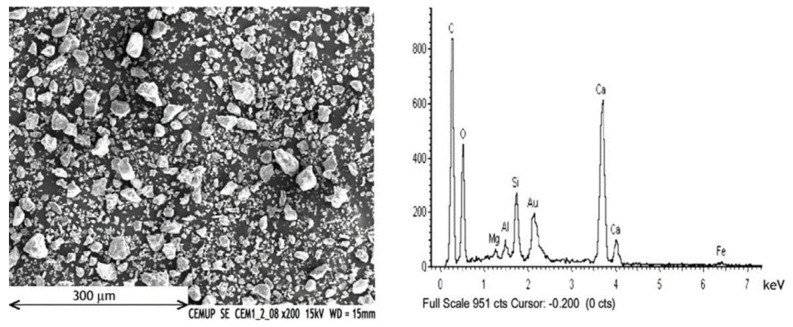
SEM micrograph and EDX analysis of waste wood ash (used with permission from Elsevier [71]).

Garcia et al. [71] studied the microstructure properties with the help of a scan electron microscope (SEM) of ground waste wooden ash, obtained from forest regions surrounding a power plant in Portugal. From the SEM images, it was observed that ground waste wooden ash has two governing properties of particles and fibers in layers. The SEM micrographs of the ground waste wooden ash at higher magnification and electron dispersive X-ray (EDX) spectra are shown in Figure 5.

Awolusi et al. [87] investigated the microstructure characteristics of OPC mortar with waste wood ash from sawdust. The mortars were developed with WWA ranging from 0 to 10% with a water to binder ratio of 0.6. SEM micrograph displayed maximum inter-spatial distance between the particles of WWA in comparison to the binder, which was dense with each other [87]. The SEM outcomes of a composite made from corn cob–polypropylene–WWA displayed that the pores present in matrix become smaller as the proportion of WWA was raised. This can be attributed to the pores between the corn cob and polypropylene being filled by the waste wooden ash. Due to the increased dose of waste wooden ash in the mix, the stress concentration stretched, and the shape of WWA was revealed to be considerably small; thus, the distances became very less. The bridging behavior of waste wooden ash could lead to a maximum wrap among corn cobs [88].

**Figure 6:**
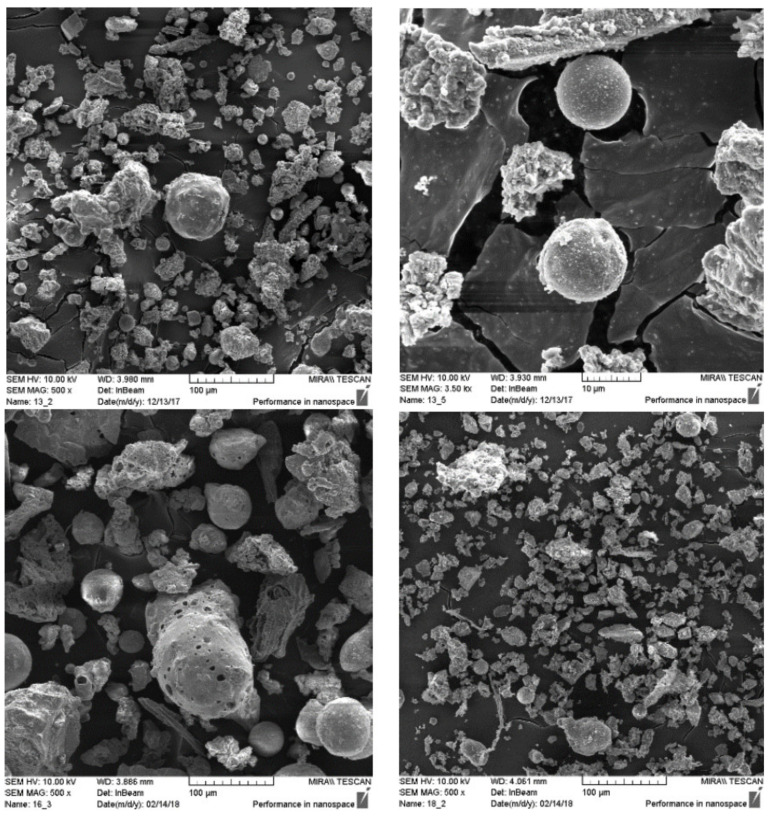
Pores in the reference concrete and reference sample with different proportions of WWA and SF (used from an open source journal of MDPI [91]).

It can be observed from the SEM image shown in Figure 6 that waste wood ash with silica fume enhanced the matrix when the ash was dispersed uniformly. Figure 6 shows the study of pores enclosed in the reference sample and sample with different proportions of waste wood ash and silica fume. The doses of waste wood ash and silica fume added to the mix considerably impacted the pores shaped in concrete mortars. The proof of this effect is displayed in the formation of larger pores in the reference specimen. Concrete mixes utilized different proportions of silica fume and waste wood ash by substituting the binder with 15% reduced estimate of pores. This positive test outcome was due to the rich silica in the silica fumes [76].

**Figure 7:**
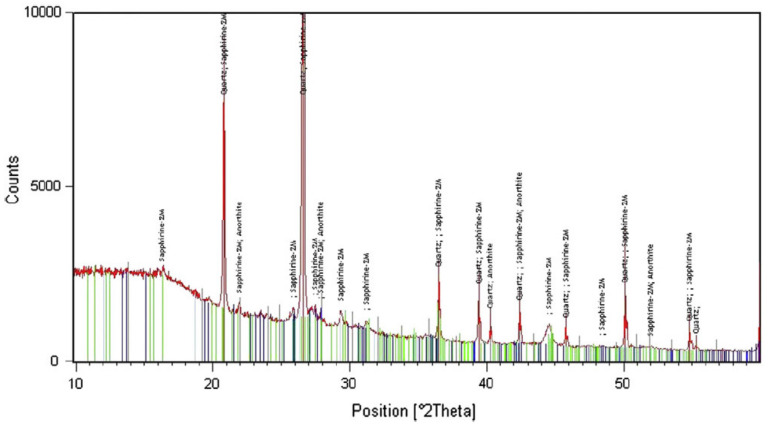
XRD analysis of waste wood ash (used with the permission from Elsevier [71]).

Chowdhury et al. [80] studied the X-ray diffraction of waste wood ash concrete. Figure 7 displays the X-ray diffraction spectra of waste wood ash concrete. The hump specifies that the specimen was formless, with the peaks of silica demonstrating a crystalline behavior. Thus, waste wood ash comprises silica in crystalline and formless shapes. Crystals of silica present high peaks at 29 degrees at 2-theta. Formless silica concentrates in the mixture as a suitable binder substitution material due to its pozzolanic behavior [80]. Similar observations were also noted by Elahi et al. [89] from the X-ray diffraction spectra of waste wood ash, which displayed the existence of formless silica, although only in low proportions. A study of the waste wood ash concrete microstructure showed that the inclusion of waste wood ash impacted the pore estimations, and a significant reduction in porosity occurred [90]. The succeeding samples showed a microstructure that was very dense with a lower permeability [90].

**Figure 8:**
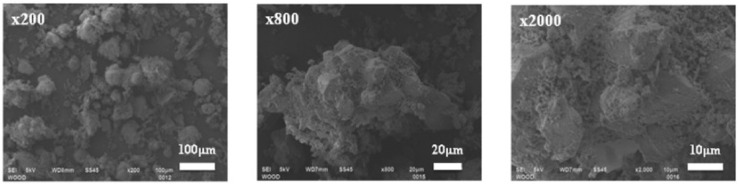
SEM image of WWA (Grau et al., 2015) (Used from an open source journal of MDPI [61]).

WWA consists of particles sizes that vary between 10 µm and 200 µm, as is observed in Figure 8. The shape and size of particles vary for WWA with small particles adhering to the surface, as is observed. WWA consists of particles with an irregular shape due to the inorganic particles present in it (Etiegni and Campbell, 1991) [62].

## 7. Effect of WWA Concrete on the Environment

WWA can have a positive influence in developing cement from an environmental standpoint [92]. The mineralogical and physical assembly of burning ash, such as WWA ash, and the availability of metals depend on the treatment of temperature and feed material [93]. The last method for using waste wood ash must be appropriately sustained due to the fineness of particles and lenience of air pollution, which can lead to breathing issues for the public near the manufacturing places [50]. In addition to this impact, the impact of waste wood ash on acidic material can result in releasing heavy metals into the environment [67]. Thus, issues have to be raised and more studies must be performed on the environmental impacts of waste wood ash concrete. Udoeyo et al. [67] utilized waste wood ash as a mineral material to research its effect on the environment and revealed that, if the waste wood ash is discarded in lands, then acidic rain releases heavy metals to the surrounding area. Thus, the usage of waste wood ash decreases pollution by reducing the necessity of discarding [73].

Gorpade et al. [72] assessed the impact of the inclusion of waste wooden ash from 0% to 30% in concrete. It was revealed that up to 10% of cement can be efficiently substituted with waste wood ash. If waste wood ash is utilized as a mineral material in concrete, the amount of discarded WWA and its adverse effects on the environment can be diminished significantly. Adding waste wood ash as a substitute for cement decreases the usage of cement in concrete, which reduces the manufacturing cost of binders and its related outflow of harmful gases. Waste wood ash is a very fine material compared to cement and it can fill all the voids within the microstructure of concrete, which makes it hard for the outside chlorides or salts to enter the concrete. Making concrete buildings near the sea areas with waste wood ash concrete is recommended to avert structure catastrophes from heavy salt-oriented climates [94].

Waste wood ash seems to be an auspicious pozzolanic material for the partial replacement of binder, with no reduction in concrete strength, with enhanced durability of sample, and contributing significantly to the sustainability of the construction industry [68]. Waste wood ash in samples help to makes an eco-efficient substitute cementitious material, which is efficient and cost-friendly [80]. Studies [70,95] have been performed to evaluate the creation of sustainable construction material by providing waste wood ash as a binder replacement material. The natural influence of the usage of waste wood ash in cement mortar, carbon impression, and the degree of consumed energy was taken as a major parameters that can be used as a quantitative limitation to signify the likely recompences of waste wood ash applications in cementitious materials. The study was carried out according to the exclusive method shown by Pavlikova et al. [69]. Waste wood ash can be an actual pozzolanic additive used as a partial substitute of cement to assist in the environmentally friendly concrete construction of buildings.

## 8. Conclusions

The quality and quantity of waste wood ash are dependent on different features, specifically, the temperature of the burning of waste wood and the type of burning technique utilized for waste wood. Thus, the appropriate classification of WWA is obligatory before its usage as an ingredient material in the development of geopolymer concrete mixes.

- The distribution of wood ash particles is usually grainier as compared to cement. However, the specific surface of WWA is moderately smoother than that of Portland cement because of the higher irregularity in wood particles and their permeable behavior.
- The chemical arrangement of WWA differs considerably within types of trees from which the biomass of wood is obtained, but it is usually rich in CaO and SiO_2_ elements.
- Binders blended with WWA as a fractional substitute have higher initial and final times and high standard consistency. Geopolymer mixes having WWA are inclined to have a low heat of hydration.
- A considerable amount of ettringite crystals is shaped within a paste of binder upon the hydration of OPC–WWA geopolymer samples, specifically at high doses of binder replacement with WWA.
- Geopolymer mixes of mortar and concrete comprising WWA as a fractional substitution of the binder have more water requirements to obtain a desirable level of slump value in comparison to similar geopolymer mixtures with no WWA.
- The addition of WWA as fractional binder substitution in mixes of mortar and concrete at a high dose of binder substitution could lead to a steady decrease in the bulk density of hard mixes of geopolymer mortar and concrete.
- Usually, the inclusion of WWA as fractional binder substitution in the preparation of geopolymer concrete blend decreases the compression, flexural, and split tensile strength of geopolymer concrete. However, there are hopeful outcomes as the addition of WWA at a low dosage level of binder substitution truly assisted in the improvement of the compression strength of the developed mixes of geopolymer concrete. WWA as a fractional substitute for binder at a substitution level of 10% by binder weight can make geopolymer mortar or concrete, which can be produced and utilized in building applications with suitable strength and durability characteristics.
- Metakaolin can be utilized as an activator for making geopolymer concrete or mortar with WWA as a fractional substitute of binder to improve the mechanical strength of geopolymer concrete or mortar.
- Geopolymer concrete blends comprising WWA as fractional binder substitute display more resistance against rusting when exposed to strong acids in comparison to mixes with no WWA.
- Geopolymer concrete blends having more quantity of WWA as a partial substitute of binder can have a high degree of water absorption.
- The utilization of WWA as fractional replacement of binder in geopolymer concrete blends at substitution levels of up to 25% by binder weight does not have detrimental impacts on the resistance of the geopolymer concrete against chloride ion diffusion. Furthermore, the utilization of 80% fly ash and 20% WWA in geopolymer concrete considerably improves the sample’s capability to resist chloride ions diffusion.
- The advantage of present study is that the addition of very fine size WWA, made from burning of rice husk, wood, bagasse, assists considerably in enhancing the durability characteristics of the geopolymer sample in terms of ASR and resistance against chloride ions. The existence of WWA in geopolymer concrete had a considerable role, as it reduced the extent of the geopolymer sample’s drying shrinkage considerably.
- The disadvantage of present study is that, to make WWA, it needs a considerable higher degree of fire to burn the waste wood, which will need a lot of energy and resources, and finding naturally burnt WWA is highly difficult.

## 9. Recommendations

Waste wood ash has the possibility of being a substitute construction material for sustainability purposes, as a fractional substitute of binder and aggregates. The usage of waste wood ash in large volumes is conceivable. Some research has been conducted on this and some hopeful outcomes have been observed, as WWA can be utilized as an eco-efficient material with little to no compromise on the properties of geopolymer concrete samples. However, for now, WWA has been utilized in a limited amount in the development of samples. This extensive review study of WWA on geopolymer concrete as a fractional substitute of binder showed that the shape, size, source, method of making WWA, and chemical and physical composition of WWA have a significant impact on the strength and durability properties of the sample in which WWA is utilized. Thus, waste wood ash is suitable as a binder replacement, and if it is used in construction as a building material, it will reduce the demand for cement, which will reduce the outflow of greenhouse gases from the production of cement and also preserve the natural reserves of limestone used in the making of cement, thus helping the environment and assisting the construction industry by increasing its sustainability.

## Figures and Tables

**Table 1:** Chemical elements of the ashes (ppm) [53].

Type of Ash	P	K	Ca	S	Cu	Fe	Mn	Zn	Ni	Cr	Pb	As
Birch wood	20,853	71,290	132,583	5631	97.10	6518	17,585	212.67	34.91	39.07	40.48	1.01
Pine wood	18,618	116,436	201,109	7142	196	3665	10,693	193.13	45.84	62.04	28.89	1.59
Oak wood	15,071	57,331	156,738	5107	190.67	9256	10,114	169.33	125.67	89.87	54.49	1.91
Horen beam wood	16,548	69,905	249,050	3956	140.67	8598	18,587	155.0	158.67	10.65	40.20	1.13
Ash wood	17,967	70,442	279,785	3077	121.00	5758	10,545	183.0	24.84	30.66	15.31	0.78
Wood residue chips—forest	17,680	69,104	203,935	1546	188.0	3403	6920	171.0	110.33	95.64	50.67	1.44
Wood residue chips—municipal	32,039	108,081	245,075	8464	181.0	4678	2815	320.33	176.33	25	12.69	0.13
Poplar wood	6419	64,985	173,872	5015	96.92	4612	549.67	81.41	26.19	20.57	9.65	0.18
Willow	3342	37,339	135,981	4732	123.5	2662	910	394.0	32.0	45.97	8.93	0.34
Acacia wood	2679	38,799	227,225	1826	158.0	6156	794.3	244.0	59.72	36.31	15.83	0.49
Average (%)	15,121.6	70,371.2	200,535.3	4649.6	149.286	5530.6	7951.297	212.387	79.45	45.578	27.714	0.9

## Data Availability

Data can be provided upon request from the corresponding author.

## References

[1] Zaid O., Mukhtar F.M., García R.M., El Sherbiny M.G., Mohamed A.M. (2022). Characteristics of high-performance steel fiber reinforced recycled aggregate concrete utilizing mineral filler. Case Stud. Constr. Mater..

[2] Althoey F., Zaid O., de-Prado-Gil J., Palencia C., Ali E., Hakeem I., Martínez-García R. (2022). Impact of sulfate activation of rice husk ash on the performance of high strength steel fiber reinforced recycled aggregate concrete. J. Build. Eng..

[3] Zaid O., Zamir Hashmi S.R., Aslam F., Alabduljabbar H. (2021). Experimental Study on Mechanical Performance of Recycled Fine Aggregate Concrete Reinforced With Discarded Carbon Fibers. Front. Mater..

[4] Zaid O., Ahmad J., Siddique M.S., Aslam F. (2021). Effect of Incorporation of Rice Husk Ash Instead of Cement on the Performance of Steel Fibers Reinforced Concrete. Front. Mater..

[5] Aslam F., Zaid O., Althoey F., Alyami S.H., Qaidi S.M.A., de Prado Gil J., Martínez-García R. (2022). Evaluating the influence of fly ash and waste glass on the characteristics of coconut fibers reinforced concrete. Struct. Concr..

[6] Zaid O., Ahmad J., Siddique M.S., Aslam F., Alabduljabbar H., Khedher K.M. (2021). A step towards sustainable glass fiber reinforced concrete utilizing silica fume and waste coconut shell aggregate. Sci. Rep..

[7] Kaza S., Lisa Y., Perinaz B.-T., Van Woerden F. (2018). What a Waste 2.0: A Global Snapshot of Solid Waste Management to 2050.

[8] Lim J., Raman S.N., Lai F.-C., Mohd Zain M.F., Hamid R. (2017). Synthesis of Nano Cementitious Additives from Agricultural Wastes for the Production of Sustainable Concrete. J. Clean. Prod..

[9] Malhotra V.M. (2002). Introduction: Sustainable Development and Concrete Technology. Concr. Int..

[10] Hh M., Al-Sulttani A., Abbood I., Hanoon A. (2020). Emissions Investigating of Carbon Dioxide Generated by the Iraqi Cement Industry. IOP Conf. Ser. Mater. Sci. Eng..

[11] Tripathi N., Hills C., Singh R., Singh J.S. (2020). Offsetting anthropogenic carbon emissions from biomass waste and mineralised carbon dioxide. Sci. Rep..

[12] Abdulkareem O.A., Matthews J., Abdullah M.M.A.B. (2018). Strength and Porosity Characterizations of Blended Biomass Wood Ash-fly Ash-Based Geopolymer Mortar. AIP Conf. Proc..

[13] Davidovits J. (2008). Geopolymer Chemistry and Applications.

[14] Ekinci E., Kazancoglu Y., Mangla S.K. (2020). Using system dynamics to assess the environmental management of cement industry in streaming data context. Sci. Total Environ..

[15] De Rossi A., Simão L., Ribeiro M., Hotza D., Moreira R. (2020). Study of cure conditions effect on the properties of wood biomass fly ash geopolymers. J. Mater. Res. Technol..

[16] Maglad A.M., Zaid O., Arbili M.M., Ascensão G., Șerbănoiu A.A., Grădinaru C.M., García R.M., Qaidi S.M.A., Althoey F., de Prado-Gil J. (2022). A Study on the Properties of Geopolymer Concrete Modified with Nano Graphene Oxide. Buildings.

[17] Zaid O., Martínez-García R., Abadel A.A., Fraile-Fernández F.J., Alshaikh I.M.H., Palencia-Coto C. (2022). To determine the performance of metakaolin-based fiber-reinforced geopolymer concrete with recycled aggregates. Arch. Civ. Mech. Eng..

[18] He X., Yuhua Z., Qaidi S., Isleem H.F., Zaid O., Althoey F., Ahmad J. (2022). Mine tailings-based geopolymers: A comprehensive review. Ceram. Int..

[19] Qaidi S.M.A., Mohammed A.S., Ahmed H.U., Faraj R.H., Emad W., Tayeh B.A., Althoey F., Zaid O., Sor N.H. (2022). Rubberized geopolymer composites: A comprehensive review. Ceram. Int..

[20] Ismail I., Bernal S.A., Provis J.L., San Nicolas R., Hamdan S., van Deventer J.S.J. (2014). Modification of phase evolution in alkali-activated blast furnace slag by the incorporation of fly ash. Cem. Concr. Compos..

[21] Salih A.P.D.M., Ali A., Farzadnia N. (2014). Characterization of mechanical and microstructural properties of palm oil fuel ash geopolymer cement paste. Constr. Build. Mater..

[22] Cheah C., Ken P., Ramli M. (2015). The hybridizations of coal fly ash and wood ash for the fabrication of low alkalinity geopolymer load bearing block cured at ambient temperature. Constr. Build. Mater..

[23] Li Z., Ding Z., Zhang Y. Development of sustainable cementitious materials. Proceedings of the International Workshop on Sustainable development and Concrete Technology.

[24] Jamil M., Khan M.N.N., Karim M., Kaish A.B.M., Zain M.F.M. (2016). Physical and chemical contributions of Rice Husk Ash on the properties of mortar. Constr. Build. Mater..

[25] Sales A., Bessa S. (2010). Use of Brazilian sugarcane bagasse ash in concrete as sand replacement. Waste Manag..

[26] Payá J., Monzo J., Borrachero M., Díaz-Pinzón L., Ordonez L.M. (2002). Sugar-cane bagasse ash (SCBA): Studies on its properties for reusing in concrete production. J. Chem. Technol. Biotechnol..

[27] Adesanya D.A. (1996). Evaluation of blended cement mortar, concrete and stabilized earth made from ordinary Portland cement and corn cob ash. Constr. Build. Mater..

[28] Rangasamy G., Mani S., Senathipathygoundar Kolandavelu S.K., Alsoufi M.S., Mahmoud Ibrahim A.M., Muthusamy S., Panchal H., Sadasivuni K.K., Elsheikh A.H. (2021). An extensive analysis of mechanical, thermal and physical properties of jute fiber composites with different fiber orientations. Case Stud. Therm. Eng..

[29] El-Kassas A., Elsheikh A.H. (2020). A new eco-friendly mechanical technique for production of rice straw fibers for medium density fiberboards manufacturing. Int. J. Environ. Sci. Technol..

[30] Elsheikh A.H., Panchal H., Shanmugan S., Muthuramalingam T., El-Kassas A.M., Ramesh B. (2022). Recent progresses in wood-plastic composites: Pre-processing treatments, manufacturing techniques, recyclability and eco-friendly assessment. Clean. Eng. Technol..

[31] Elsheikh A.H., Abd Elaziz M., Ramesh B., Egiza M., Al-Qaness M.A.A. (2021). Modeling of drilling process of GFRP composite using a hybrid random vector functional link network/parasitism-predation algorithm. J. Mater. Res. Technol..

[32] Showaib E.A., Elsheikh A.H. (2020). Effect of surface preparation on the strength of vibration welded butt joint made from PBT composite. Polym. Test..

[33] Anand Raj M.K., Muthusamy S., Panchal H., Mahmoud Ibrahim A.M., Alsoufi M.S., Elsheikh A.H. (2022). Investigation of mechanical properties of dual-fiber reinforcement in polymer composite. J. Mater. Res. Technol..

[34] Danraka M., Aziz F., Jaafar M., Mohd Nasir N., Abdulrashid S. Application of Wood Waste Ash in Concrete Making: Revisited. Proceedings of the Global Civil Engineering Conference (GCEC 2017).

[35] Candamano S., De Luca P., Frontera P., Crea F. (2017). Production of Geopolymeric Mortars Containing Forest Biomass Ash as Partial Replacement of Metakaolin. Environments.

[36] Ekaputri J.J., Triwulan Damayanti O. (2015). The Influence of Alkali Activator Concentration to Mechanical Properties of Geopolymer Concrete with Trass as a Filler. Mater. Sci. Forum.

[37] Ekaputri J. (2015). Geopolymer Grout Material. Mater. Sci. Forum.

[38] Smirnova O., Menéndez-Pidal I., Alekseev A., Petrov D., Popov M. (2022). Strain Hardening of Polypropylene Microfiber Reinforced Composite Based on Alkali-Activated Slag Matrix. Materials.

[39] Smirnova O. (2018). Development of classification of rheologically active microfillers for disperse systems with Portland cement and superplasticizer. Int. J. Civ. Eng. Technol..

[40] Smirnova O.M. (2020). Low-Clinker Cements with Low Water Demand. J. Mater. Civ. Eng..

[41] Smirnova O.M., de Navascués I., Mikhailevskii V.R., Kolosov O.I., Skolota N.S. (2021). Sound-Absorbing Composites with Rubber Crumb from Used Tires. Appl. Sci..

[42] Yakovlev G., Polyanskikh I., Gordina A., Pudov I., Černý V., Gumenyuk A., Smirnova O. (2021). Influence of Sulphate Attack on Properties of Modified Cement Composites. Appl. Sci..

[43] Saidova Z., Yakovlev G., Smirnova O., Gordina A., Kuzmina N. (2021). Modification of Cement Matrix with Complex Additive Based on Chrysotyl Nanofibers and Carbon Black. Appl. Sci..

[44] Smirnova O., Kazanskaya L., Koplík J., Tan H., Gu X. (2021). Concrete Based on Clinker-Free Cement: Selecting the Functional Unit for Environmental Assessment. Sustainability.

[45] Smirnova O. (2019). Compatibility of shungisite microfillers with polycarboxylate admixtures in cement compositions. ARPN J. Eng. Appl. Sci..

[46] Alves L., Leklou N., de Barros S. (2020). A comparative study on the effect of different activating solutions and formulations on the early stage geopolymerization process. MATEC Web Conf..

[47] Kumar A., Muthukannan M., Babu A., Hariharan A., Muthuramalingam T. (2020). Effect on addition of Polypropylene fibers in wood ash-fly ash based geopolymer concrete. IOP Conf. Ser. Mater. Sci. Eng..

[48] Kristály F., Szabo R., Madai F., Ákos D., Mucsi G. (2020). Lightweight composite from fly ash geopolymer and glass foam. J. Sustain. Cem. Mater..

[49] Ali B., Raza S., Kurda R., Alyousef R. (2021). Synergistic effects of fly ash and hooked steel fibers on strength and durability properties of high strength recycled aggregate concrete. Resour. Conserv. Recycl..

[50] Aprianti E., Shafigh P., Bahri S., Farahani J.N. (2015). Supplementary cementitious materials origin from agricultural wastes—A review. Constr. Build. Mater..

[51] Pitman R. (2006). Wood ash use in forestry—A review of the environmental impacts. Forestry.

[52] Kurda R., de Brito J., Silvestre J.D. (2017). Influence of recycled aggregates and high contents of fly ash on concrete fresh properties. Cem. Concr. Compos..

[53] Zając G., Szyszlak-Bargłowicz J., Gołębiowski W., Szczepanik M. (2018). Chemical Characteristics of Biomass Ashes. Energies.

[54] Kahl J., Fernandez I., Rustad L., Peckenham J. (1996). Threshold Application Rates of Wood Ash to an Acidic Forest Soil. J. Environ. Qual..

[55] Fransman B., Nihlgård B.J. (1995). Water chemistry in forested catchments after topsoil treatment with liming agents in South Sweden. Water. Air. Soil Pollut..

[56] Eriksson J. (1996). Dissolution of Hardened Wood Ash in Forest Soils Studies in a Column Experiment.

[57] Baker A., Alloway B.J. (1995). Heavy Metals in Soils.

[58] Lanzerstorfer C. (2015). Chemical composition and physical properties of filter fly ashes from eight grate-fired biomass combustion plants. J. Environ. Sci..

[59] Williams T.M., Hollis C.A., Smith B.R. (1996). Forest Soil and Water Chemistry following Bark Boiler Bottom Ash Application. J. Environ. Qual..

[60] Steenari B.-M., Karlsson L.G., Lindqvist O. (1999). Evaluation of the leaching characteristics of wood ash and the influence of ash agglomeration. Biomass Bioenergy.

[61] Grau F., Choo H., Hu J.W., Jung J. (2015). Engineering Behavior and Characteristics of Wood Ash and Sugarcane Bagasse Ash. Materials.

[62] Etiégni L., Campbell A.G. (1991). Physical and chemical characteristics of wood ash. Bioresour. Technol..

[63] Lanzerstorfer C. (2017). Fly Ash from the Combustion of Post-Consumer Waste Wood: Distribution of Heavy Metals by Particle Size. Int. J. Environ. Sci..

[64] Szakova J., Ochecova P., Hanzlicek T., Perna I., Tlustos P. (2013). Viability of total and mobile element contents in ash derived from biomass combustion. Chem. Pap..

[65] Karltun E., Saarsalmi A., Ingerslev M., Mandre M., Andersson S., Gaitnieks T., Ozolinčius R., Varnagiryte-Kabasinskiene I. (2008). Wood Ash Recycling—Possibilities And Risks. Sustainable Use of Forest Biomass for Energy.

[66] Abdullahi M. (2006). Characteristics of wood ash/OPC concrete. Leonardo Electron. J. Pract. Technol..

[67] Udoeyo F., Inyang H., Young D., Oparadu E. (2006). Potential of Wood Waste Ash as an Additive in Concrete. J. Mater. Civ. Eng..

[68] Ramos T., Matos A.M., Sousa-Coutinho J. (2013). Mortar with wood waste ash: Mechanical strength carbonation resistance and ASR expansion. Constr. Build. Mater..

[69] Pavlíková M., Zemanová L., Pokorny J., Záleská M., Jankovský O., Lojka M., Sedmidubský D., Pavlik Z. (2018). Valorization of wood chips ash as an eco-friendly mineral admixture in mortar mix design. Waste Manag..

[70] Chowdhury S., Mishra M., Suganya O. (2015). The incorporation of wood waste ash as a partial cement replacement material for making structural grade concrete: An overview. Ain Shams Eng. J..

[71] Da Luz Garcia M., Sousa-Coutinho J. (2013). Strength and durability of cement with forest waste bottom ash. Constr. Build. Mater..

[72] Ghorpade V.G. (2012). Effect of wood waste ash on the strength characteristics of concrete. Nat. Environ. Pollut. Technol..

[73] Horsakulthai V., Phiuvanna S., Kaenbud W. (2011). Investigation on the corrosion resistance of bagasse-rice husk-wood ash blended cement concrete by impressed voltage. Constr. Build. Mater..

[74] Sashidhar C., Rao S. Durability Studies On Concrete with Wood Ash Additive. Proceedings of the 35th Conference on Our World in Concrete & Structures.

[75] Okeyinka O.M., Oladejo O.A. (2014). The Influence of Calcium Carbonate as an Admixture on the Properties of Wood Ash Cement Concrete. Int. J. Emerg. Technol. Adv. Eng..

[76] Mydin M.A., Shajahan M.F., Ganesan S., Md Sani N. (2014). Laboratory Investigation on Compressive Strength and Micro-structural Features of Foamed Concrete with Addition of Wood Ash and Silica Fume as a Cement Replacement. MATEC Web Conf..

[77] Kusuma S. (2015). Studies on strength characteristics of fibre reinforced concrete with wood waste ash. Int. Res. J. Eng. Technol..

[78] Prabagar S., Subasinghe K., Fonseka W. (2015). Wood ash as an effective raw material for concrete blocks. Int. J. Res. Eng. Technol..

[79] Fapohunda C., Bolatito A., Akintoye O. (2018). A Review of the Properties, Structural Characteristics and Application Potentials of Concrete Containing Wood Waste as Partial Replacement of one of its Constituent Material. YBL J. Built Environ..

[80] Chowdhury S., Maniar A., Suganya O.M. (2015). Strength development in concrete with wood ash blended cement and use of soft computing models to predict strength parameters. J. Adv. Res..

[81] Udoeyo F., Dashibil P. (2002). Sawdust Ash as Concrete Material. J. Mater. Civ. Eng..

[82] Elinwa A.U., Ejeh S.P. (2004). Effects of the Incorporation of Sawdust Waste Incineration Fly Ash in Cement Pastes and Mortars. J. Asian Archit. Build. Eng..

[83] Wang S., Llamazos E., Baxter L., Fonseca F. (2008). Durability of biomass fly ash concrete: Freezing and thawing and rapid chloride permeability tests. Fuel.

[84] (2009). Standard Test Method for Electrical Induction of Concrete, Stability to Resist Chloride Ion Penetration.

[85] Wang S., Baxter L. (2007). Comprehensive study of biomass fly ash in concrete: Strength, microscopy, kinetics and durability. Fuel Process. Technol..

[86] Naik T., Kraus R.N., Siddique R. (2003). Controlled low-strength materials containing mixtures of coal ash and new pozzolanic material. ACI Mater. J..

[87] Awolusi T.F., Sojobi A.O., Afolayan J.O. (2017). SDA and laterite applications in concrete: Prospects and effects of elevated temperature. Cogent Eng..

[88] Wan Y., Wu H., Huang L., Zhang J., Tan S., Cai X. (2018). Preparation and characterization of corn cob/polypropylene composite reinforced by wood ash. Polym. Bull..

[89] Elahi M., Qazi A., Yousaf M., Akmal U. (2015). Application of wood ash in the production of concrete. Sci. Int..

[90] Cheah C.B., Ramli M. (2011). The implementation of wood waste ash as a partial cement replacement material in the production of structural grade concrete and mortar: An overview. Resour. Conserv. Recycl..

[91] Gabrijel I., Jelčić Rukavina M., Štirmer N. (2021). Influence of Wood Fly Ash on Concrete Properties through Filling Effect Mechanism. Materials.

[92] Yin K., Ahamed A., Lisak G. (2018). Environmental perspectives of recycling various combustion ashes in cement production—A review. Waste Manag..

[93] Vollprecht D., Berneder I., Capo Tous F., Stöllner M., Sedlazeck P., Schwarz T., Aldrian A., Lehner M. (2018). Stepwise treatment of ashes and slags by dissolution, precipitation of iron phases and carbonate precipitation for production of raw materials for industrial applications. Waste Manag..

[94] Manikanta B., Vummaneni R.R., Achyutha Kumar Reddy M. (2018). Performance of wood ash blended reinforced concrete beams under acid (HCl), base (NaOH) and salt (NaCl) curing conditions. Int. J. Eng. Technol..

[95] Siddique R. (2012). Utilization of wood ash in concrete manufacturing. Resour. Conserv. Recycl..

